# Clinicopathological characteristics of non-diabetic renal disease in patients with type 2 diabetes mellitus in a northeastern Chinese medical center: a retrospective analysis of 273 cases

**DOI:** 10.1007/s11255-016-1331-y

**Published:** 2016-06-06

**Authors:** Shujun Liu, Qiaoyan Guo, Hongbo Han, Peihe Cui, Xiao Liu, Lining Miao, Hongbin Zou, Guangdong Sun

**Affiliations:** 1 The Department of Nephrology, Second Hospital of Jilin University, Changchun, 130041 Jilin China; 2 The Department of Endocrinology, 208th Hospital of PLA, Changchun, China

**Keywords:** Non-diabetic renal disease, Diabetic nephropathy, Clinical manifestations, Type 2 diabetes, Renal biopsy, Histopathology

## Abstract

**Objective:**

To investigate the clinical and histopathological features of non-diabetic renal disease (NDRD) superimposed on diabetic nephropathy (DN) in northeastern Chinese patients with type 2 diabetes mellitus (T2D), and compare the changes with those of pure DN and isolated NDRD.

**Methods:**

Single-center retrospective analysis based on medical records of 273 patients (172 men, mean age: 51.1 ± 12.4 years) with T2D who underwent renal biopsy between February 2000 and October 2015. All patients were diagnosed as cases of pure DN, isolated NDRD or NDRD superimposed on DN.

**Results:**

Out of the 273 T2D patients, 68 (24.9 %) had DN, 175 (64.1 %) had NDRD, and 30 (11.0 %) had NDRD superimposed on DN. Idiopathic membranous nephropathy (IMN, 29.7 %) was the most common NDRD followed by IgA nephropathy (IgAN, 22.9 %), and hypertensive renal arteriolar sclerosis was the most common lesion in patients diagnosed as NDRD superimposed on DN. Patients with NDRD had a shorter duration of diabetes and lower frequencies of diabetic retinopathy (DR, 6.9 %) and renal failure (28.0 %), which is consistent with higher estimated glomerular filtration rates (eGFR) and lower systolic blood pressure (SBP). No significant between-group differences were observed with respect to proteinuria and hematuria.

**Conclusion:**

Renal biopsy is strongly recommended for T2D patients to distinguish DN, NDRD and NDRD superimposed on DN, especially in patients with no signs of DR. This approach may help in early diagnosis and treatment of NDRD and improve renal outcomes in northeastern Chinese T2D patients.

## Introduction

With the rising prevalence of diabetes mellitus (DM), diabetic nephropathy (DN) is now the most common cause of end stage-renal disease (ESRD) worldwide [1]. The condition imposes a heavy economic burden on the healthcare system in China [2]. Type 2 diabetes is associated with more heterogeneous renal lesions than those in type 1 diabetes [2–5]. Moreover, non-diabetic renal disease such as IgA nephropathy (IgAN), membranous nephropathy (MN) and mesangioproliferative glomerulonephritis (MPGN) may coexist with DN. Differentiation between DN and non-diabetic renal disease (NDRD) may not be possible without renal biopsy.

DN is typically irreversible, while NDRDs may be amenable to cure with early diagnosis and treatment; therefore, management and prognosis of NDRD and DN are quite different. Identification of NDRD with renal biopsy may help guide specific alteration in treatment and, thereby, improve prognosis [6, 7]. The indications for renal biopsy in type 2 diabetic patients with NDRD include a recent history of DM, lower HbA1c levels, and normal blood pressure (BP), absence of DR, rapid decline in renal function, increasing proteinuria, active urine sediment or acute onset of nephrotic syndrome (NS) [6, 8, 9].

Occurrence of NDRD in type 2 diabetic patients has been increasingly documented in recent years [10–12]; prevalence of NDRD shows considerable regional variations worldwide [2, 6, 13]. The estimated prevalence of NDRD among Chinese type 2 diabetic patients is between 13.4 and 82.9 % [14–18]. The global prevalence of NDRD superimposed on DN is believed to be less than 50 % [2], yet the prevalence of NDRD combined with DN in northeastern China is not clear.

In this retrospective, single-center study, we assessed the prevalence of DN alone, isolated NDRD, and NDRD superimposed on DN in type 2 diabetic patients. Detailed analysis of clinical and histopathological features of NDRD with or without DN in T2D patients is presented to characterize the different nature of NDRD in northeastern China.

## Materials and methods

### Patients

The present study was conducted in the Department of Nephrology, Second Hospital of Jilin University, in northeastern China. Of the 4830 patients who underwent renal biopsy from February 2000 to October 2015, 311 type 2 diabetic patients were identified based on the diagnostic criteria of the American Diabetic Association [19]. After exclusion of patients with malignancies and those with history of kidney transplantation and secondary DM, 273 patients for whom sufficient clinical and laboratory data were available were enrolled in this study. The indications for renal biopsy included unexplained rapid increase in proteinuria or decline in renal function, persistent hematuria of glomerular origin, acute onset of NS, renal involvement in the absence of DR, and patients with recent history of DM. The study protocol was approved by the Human Ethics Review Committee at the Second Hospital of Jilin University; written informed consent was obtained from all patients prior to renal biopsy.

### Data collection

Data on the following demographic and clinical variables were collected at, or close to the time of renal biopsy: age, sex, duration of DM, presence of DR, hypertension, dyslipidemia, NS, renal failure, proteinuria and hematuria, body mass index (BMI), blood pressure, kidney size (kidneys mean maximal longitudinal axis on abdominal ultrasonography), and treatment history with insulin and/or renin–angiotensin–aldosterone system (RAAS) blockade.

Data on following laboratory parameters were collected: hemoglobin, serum albumin, creatinine and total cholesterol levels, glycated hemoglobin (HbA1c), and 24-h urine protein excretion. Viral markers (HBsAg and anti-hepatitis C virus or HIV) were investigated in all cases. Estimated glomerular filtration rate (eGFR in mL/min/1.73 m^2^) was calculated from serum creatinine level using the Modified Diet in Renal Disease (MDRD) study equation [37].

Proteinuria was defined as >0.3 g/24 h. NS was described as proteinuria (>3.5 g/24 h) accompanied by hypoalbuminemia (<30 g/L), edema, and hyperlipidemia. Hematuria was defined as >3 red blood cells per high-power field on urine examination. Renal failure was defined as serum creatinine >178 μmol/L. Hypertension was defined as a systolic BP > 140 mmHg and/or diastolic BP > 90 mmHg.

### Renal biopsy

The preferred site for renal biopsy was the lateral aspect of the lower pole of the left kidney under ultrasound guidance with the patient in the prone position. An automated biopsy gun and a 16- or 18-gauge needle was used to ensure the biopsy sample contained a minimum of ten glomeruli. Renal biopsy specimens were prepared according to standard methods for light microscopy and immunofluorescence; electron microscopy was not routinely performed. For light microscopy, hematoxylin and eosin (HE) staining, periodic acid-Schiff’s reagent (PAS) staining, periodic Schiff-methenamine (PASM) staining, and Masson’s trichrome solution (Masson) staining were performed. In certain cases, Congo red and methyl violet staining were also done. Immunofluorescent staining for IgA, IgG, IgM, C3, C4, C1q, Fib, and κ/λ light chains was conducted [20]. All pathological diagnoses were made by the same pathologist at the Second Hospital of Jilin University.

### Pathological diagnostic criteria

DN was diagnosed based on the presence of diffuse or nodular glomerulosclerosis, mesangial (nodular or diffuse) widening, glomerular hypertrophy, glomerular capillary wall thickening, evidence of exudative lesions including fibrin caps, capsular drops, or hyaline thrombi. The diagnosis of various NDRD was made on the basis of characteristic features on light microscopy examinations in the absence of histological features of DN.

All patients were divided into three groups based on the biopsy findings: pure DN group (DN), isolated NDRD group (NDRD), and NDRD superimposed on DN group (DN + DNRD).

### Statistical analysis

Statistical analyses were performed using PRISM software (Graph Pad, San Diego, CA). Quantitative data on variables with a normal distribution are presented as mean ± standard deviation (SD); those with a skewed distribution are expressed as median (range); categorical variables were presented as frequencies and percentages [*n* (%)]. Differences between groups were assessed with *t* test or analysis of variance (ANOVA) for continuous variables. Values of *P* < 0.05 were regarded as statistically significant.

## Results

### Demographic and clinical characteristics

**Fig. 1:**
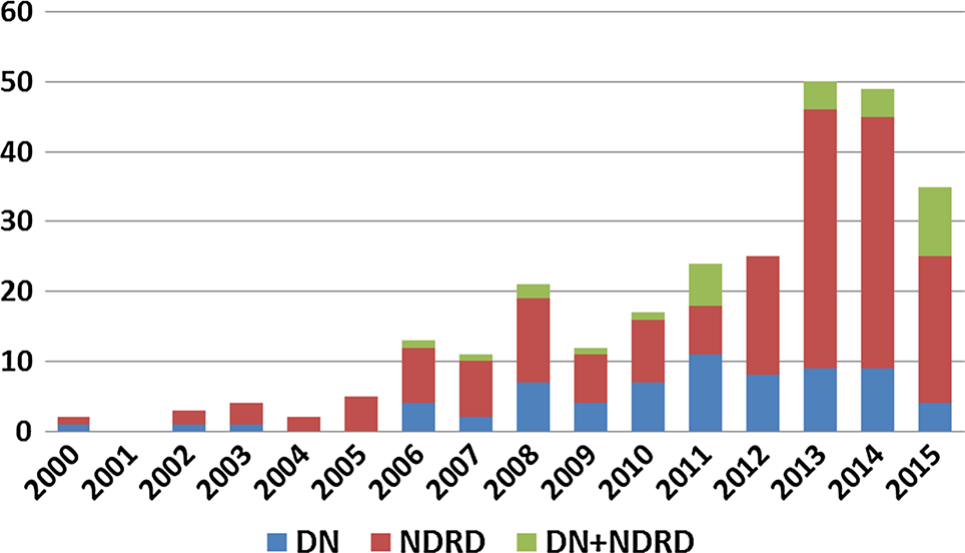
Number of type 2 diabetic patients who underwent renal biopsy between 2000 and 2015. *DN* diabetic nephropathy, *NDRD* non-diabetic renal disease, *DN* + *NDRD* NDRD superimposed on DN

Out of a total of 4830 Chinese patients who underwent renal biopsy, 273 patients with T2D were included in the present study. As shown in Fig. 1, the number of T2D patients undergoing renal biopsy has increased over the last 15 years. Out of 273 patients, 68 patients (24.9 %) had a pathological diagnosis of DN, 175 (64.1 %) had isolated NDRD, and 30 (11.0 %) had NDRD combined with DN (Table 1).

**Table 1:** Baseline characteristics of 273 type 2 diabetic patients at the time of renal biopsy

Variables	Results
Age at biopsy (years) mean ± SD (range)	51.1 ± 12.4 (22–82)
Sex (male/female)	(172/101)
Duration of DM (months)	52.0 ± 68.9 (1–487)
Insulin treatment (yes, %)	110 (40.3 %)
Diabetic retinopathy (yes, %)	37 (13.6 %)
Nephrotic syndrome (yes, %)	77 (28.2 %)
Renal failure (yes, %)	42 (15.4 %)
Hypertension (yes, %)	115 (42.1 %)
Dyslipidemia (yes, %)	188 (68.9 %)
RAAS blockade therapy (yes, %)	113 (41.4 %)
Body mass index (kg/m^2^)	25.9 ± 4.1 (16–41.1)
24 h-urinary protein (g)	4.81 ± 4.16 (0.3–28.2)
Proteinuria (%)	261 (95.6 %)
Hematuria (%)	188 (68.9 %)
*Pathological diagnosis*	
Diabetic nephropathy (DN)	68 (24.9 %)
Non-diabetic renal disease (NDRD)	175 (64.1 %)
NDRD superimposed on DN	30 (11.0 %)

*NDRD* non-diabetic renal disease, *DN* diabetic nephropathy, *DN* + *NDRD* NDRD superimposed on DN, *RAAS* renin–angiotensin–aldosterone system, *SD* standard deviation

The study population included 172 men and 101 women (male to female ratio 1.70); the mean age was 51.1 ± 12.4 years (range 22–8 years). Table 1 shows general characteristics of the study population. Duration of DM showed much variability (range 1–487 months). DR was present in 37 patients (13.6 %). A majority of patients had proteinuria and hematuria (261 cases, 95.6 % and 188 cases, 68.9 %, respectively). 77 patients (28.2 %) had NS; 42 patients (15.4 %) had renal failure; 115 patients (42.1 %) had hypertension, and 188 patients (68.9 %) had dyslipidemia.

Mean 24-h urine protein excretion was 4.81 ± 4.16 g (range 0.3–28.2). 110 patients (40.3 %) received insulin treatment; 113 patients (41.4 %) underwent RAAS blockade therapy at the time of renal biopsy.

### Pathological types of NDRD in T2D patients with or without DN

Primary glomerulonephritis (GN) was more common than secondary GN among patients with NDRD. As shown in Table 2, membranous nephropathy (MN) was the most common type of primary NDRD lesion (52 [29.7 %] patients); the second most common primary NDRD diagnosis was IgAN (40 [22.9 %] patients); other less common primary NDRD lesions were MPGN (14 patients), minimal change disease (MCD) (6 patients), crescentic GN (4 patients), and focal segmental glomerulosclerosis (FSGS) (3 patients). Interstitial nephritis (IN) accounted for 5.7 % of all cases. Lupus nephritis (LN) was the most common secondary NDRD lesion (9 [5.1 %] patients), and HBV-associated glomerulonephritis was the second common secondary NDRD lesion (8 patients); the other less common secondary lesions of NDRD included hypertensive renal arteriolar sclerosis (6 patients), ANCA-associated vasculitis (AAV) (5 patients), amyloidosis and TMA were observed in 4 patients each, and Henoch-Schonlein purpura, lipoprotein glomerulopathy, acute tubular necrosis (ATN) in 1 patient each.

**Table 2:** Pathological diagnosis of NDRD, with or without DN in type 2 diabetic patients

Pathologic diagnosis based on renal biopsy	NDRD (*N* = 175)	DN + NDRD (*N* = 30)	Total (*N* = 205)
Membranous nephropathy	52 (29.7)	6 (20)	58 (28.3)
IgA nephropathy	40 (22.9)	1 (3.3)	41 (20)
Mesangioproliferative glomerulonephritis (MPGN)	14 (8.0)	0	14 (6.8)
Minimal change disease	6 (3.4)	0	6 (2.9)
Crescentic glomerulonephritis	4 (2.3)	0	4 (2.0)
Focal segmental glomerulosclerosis	3 (1.7)	0	3 (1.5)
Interstitial nephritis	10 (5.7)	1 (3.3)	11 (5.4)
Lupus nephritis	9 (5.1)	0	9 (4.4)
HBV-associated glomerulonephritis	8 (4.6)	0	8 (3.9)
Hypertensive renal arteriolar sclerosis	6 (3.4)	19 (63.3)	25 (12.2)
ANCA-associated vasculitis	5 (2.9)	1 (3.3)	6 (2.9)
Thrombotic microangiopathy (TMA)	4 (2.3)	2 (6.7)	6 (2.9)
Amyloidosis	4 (2.3)	0	4 (2.0)
Henoch-Schonlein purpura	1 (0.6)	0	1 (0.5)
Lipoprotein glomerulopathy	1 (0.6)	0	1 (0.5)
Acute tubular necrosis	1 (0.6)	0	1 (0.5)
Others	7 (3.9)	0	7 (3.3)

*NDRD* non-diabetic renal disease, *DN* diabetic nephropathy, *DN* + *NDRD* NDRD superimposed on DN, *IgA* Immunoglobulin A, *HBV* hepatitis B virus, *ANCA* anti-neutrophil cytoplasmic antibody

The most common NDRD combined with DN was hypertensive renal arteriolar sclerosis (19 patients), MN was the second common lesion (6 patients), TMA was noted in 2 cases, and the other NDRD in this group included IgAN, AAV and interstitial nephritis (one patient each).

### Comparison of clinical and laboratory data

Table 3 shows the baseline patient demographic, clinical, and laboratory parameters. No significant between-group differences were observed with respect to age, sex, incidence of proteinuria and hematuria, BMI, serum creatinine, serum albumin, total cholesterol, HbA1c levels, and kidney long axis. A statistically significant difference was observed in baseline SBP and hemoglobin between the groups. The NDRD group showed a lower tendency for development of renal failure in as compared to DN + NDRD group. Patients in the pure DN and DN + NDRD groups had a longer duration of DM at the time of biopsy as compared to those in the isolated NDRD group; this difference was especially prominent in the subgroup of patients who had >120-month-long history of DM. Insulin treatment was similar in all the three groups. Over 23 % patients in the DN + NDRD group suffered from DR, while only 6.9 % patients in the isolated NDRD group had DR.

**Table 3:** Comparison of demographic, clinical characteristics, and laboratory data of diabetic patients by study group (*N* = 273)

Parameters	DN (*N* = 68)	NDRD (*N* = 175)	DN + NDRD (*N* = 30)
Age at biopsy (years)	50.8 ± 10.3	50.6 ± 12.9	54.9 ± 13.4
Sex (male/female)	44/24	111/64	17/13
Duration of DM (months)	87.1 ± 73.2 (1–243.3)	32.7 ± 52.1 (1–396)*	89.3 ± 98.6 (1–486.6)&
<12 months	13 (19.1 %)	88 (50.3 %)	5 (16.7 %)
12–60 months	17 (25 %)	50 (28.6 %)	7 (23.3 %)
61–120 months	11 (16.2 %)	23 (13.1 %)	8 (26.7 %)
>120 months	27 (39.7 %)	14 (8.0 %)	10 (33.3 %)
Insulin treatment (yes, %)	35 (51.5 %)	55 (31.4 %)	20 (66.7 %)
Diabetic retinopathy (yes, %)	18 (26.5 %)	12 (6.9 %)	7 (23.3 %)
Proteinuria (yes, %)	66 (97.1 %)	165 (94.3 %)	30 (100 %)
Hematuria (yes, %)	51 (80.9 %)	116 (66.3 %)	21 (70.0 %)
Body mass index (kg/m^2^)	25.2 ± 3.2 (17.3–33.3)	26.2 ± 4.4 (16–41.1)	25.9 ± 3.5 (19.5–33.0)
<18	2 (2.9 %)	5 (2.9 %)	0 (0.0 %)
18–25	30 (44.1 %)	60 (34.3 %)	8 (26.7 %)
>25	36 (53.0 %)	110 (62.8 %)	22 (73.3 %)
eGFR at Biopsy (mL/min/1.73 m^2^)	55.7 ± 3.3	74.9 ± 3.0*	61.1 ± 6.9
Renal failure (yes, %)	29 (42.6 %)	49 (28.0 %)*	13 (43.3 %)
Dyslipidemia (yes, %)	49 (72.1 %)	118 (67.4 %)	21 (70.0 %)
Hypertension (yes, %)	40 (58.8 %)	45 (25.7 %)	30 (100 %)
Systolic BP (mmHg)	153 ± 22 (120–230)	137 ± 19 (100–230)*	153 ± 20 (120–220)&
Diastolic BP (mmHg)	88 ± 11 (70–120)	87 ± 13 (60–150)	91 ± 12 (60–118)
Kidney long axis (cm)	10.9 ± 1.0 (9.4–13.8)	10.8 ± 0.9 (9.0–13.4)	10.9 ± 0.8 (9.7–12.9)
Laboratory findings			
Hemoglobin (g/L)	117 ± 22 (71–172)	133 ± 29 (30–186)*	120 ± 25 (58–174)&
Serum creatinine (μmol/L)	149.8 ± 159.2 (46.2–1154.3)	139.0 ± 158.4 (26.0–1202.2)	137.1 ± 93.0 (44.0–395.2)
Serum albumin (g/L)	30.8 ± 7.8 (15.3–51.9)	32.7 ± 9.0 (13.1–49.1)	31.6 ± 9.2 (20.0–51.6)
Total cholesterol (mmol/L)	6.6 ± 1.8 (3.4–13.1)	6.9 ± 3.1 (1.8–20.1)	7.1 ± 2.8 (3.2–15.0)
HbA1c (%)	7.4 ± 2.4 (4.4–17.7)	6.9 ± 1.9 (4.6–16.2)	6.7 ± 1.3 (3.8–9.7)
Proteinuria (g/d)	5.26 ± 3.60 (0.15–19)	4.41 ± 4.33 (0.13–28.2)	6.12 ± 4.12 (0.35–15.62)

*DN* diabetic nephropathy, *NDRD* non-diabetic renal disease, *DN* + *NDRD* NDRD superimposed on DN, *HbA1c* glycated hemoglobin

* *P* < 0.05 for comparison of NDRD versus DN subgroups *& P* < 0.05 for comparison of DN + NDRD versus NDRD subgroups

The rate of baseline heavy proteinuria (>3.5 g) was significantly higher in patients in the pure DN and DN + NDRD groups, as compared to those in the isolated NDRD group. On the contrary, the rate of severe hematuria (Urine RBC > 20/HP) was significantly higher in the NDRD group as compared to that in the DN + NDRD group. No significant difference in eGFR levels was observed between the three groups. No significant between-group differences were observed with respect to results of serological tests for systemic diseases including hepatitis B surface antigen or hepatitis C virus antibodies, and HIV (Table 4).

**Table 4:** Reported indicators and significant laboratory values in diabetic patients undergoing renal biopsy

Variables	DN (*N* = 68)	NDRD (*N* = 175)	DN + NDRD (*N* = 30)
Proteinuria (g/day)			
*Data not available*	1 (1.5)	3 (1.7)	0 (0)
<0.5	1 (1.5)	17 (9.7)	2 (6.7)
0.5–3.5	23 (33.8)	81 (46.3)	6 (20.0)
>3.5	43 (63.2)	74 (42.3)	22 (73.3)
Hematuria			
*Data not available*	3 (4.4)	1 (0.6)	1 (3.3)
Urine RBC > 3/HPF	51 (75)	117 (66.9)	21 (70)
Urine RBC > 10/HPF	21 (30.9)	61 (34.9)	11 (36.7)
Urine RBC > 20/HPF	10 (14.7)	48 (27.4)	7 (10.3)
Serum creatinine (μmol/L)			
*Data not available*	0 (0.0)	1 (0.6)	0 (0.0)
<178	57 (83.9)	148 (84.5)	25 (83.3)
≥178	11 (16.1)	26 (14.9)	5 (16.7)
Any positive serologic test			
(+) HBsAg or HCV antibody	18 (26.5)	32 (18.3)	5 (16.7)
(+) HIV	0 (0.0)	0 (0.0)	0 (0.0)

*DN* diabetic nephropathy, *NDRD* non-diabetic renal disease, *DN* + *NDRD* NDRD superimposed on DN, *HPF* high-power field, *HBsAg* hepatitis B surface antigen, *HCV* hepatitis C virus, *HIV* human immunodeficiency virus

## Discussion

We assessed the clinical and pathological characteristics of DN, NDRD, and NDRD superimposed on DN in type 2 diabetic patients in a single medical center of northeastern China; the relationship between clinical or laboratory data and renal pathological characteristics is assessed.

The number of renal biopsy performed in T2D patients of northeastern China is increasing year by year. Renal biopsies revealed that 75 % cases had NDRD among those tested; this finding is in the range of other reports (17–85 %) [6, 12, 14, 15, 17, 18, 21–26] and a high incidence of NDRD complicating DN (11.0 %). A variety of renal lesions can occur in T2D patients, the most common primary pathological types in NDRD group was MN (29.7 %), followed by IgAN (22.9 %), and the most secondary pathologic type was LN (5.1 %) in this group. This finding is consistent with Asian results reported earlier [6, 13, 27]. In the DN + NDRD group, all patients had pathologic hallmarks of DN and the most common NDRD lesion was hypertensive renal arteriolar sclerosis (63.3 % of all cases) followed by MN (20 %). Overall, the common lesions in NDRD and DN + NDRD groups were MN (28.3 %), IgAN (20 %), hypertensive renal arteriolar sclerosis (12.2 %), MPGN (6.8 %), and interstitial nephritis. Our findings differ from those reported earlier in southeastern Asia, in which IgAN was the most common NDRD (up to 65 %) [6, 21, 22, 26]. In contrast, FSGS have been proven to be the most common pathological type in USA [28], New Zealand [9], and Croatia [29], while AIN was the most prevalent pathological type in India, Taiwan, and Malaysia [6, 25, 26, 30]. A study showed hypertensive renal damage to be the most common pathology in China [18], and IgAN is the most common lesion in NDRD superimposed on DN [2, 17] in another medical center. The disease spectrum of NDRD with or without DN in northeastern China may differ from that in other parts of the world. Hereditary and racial predisposition to different glomerulopathies, plus different eligibility criteria for renal biopsy in T2D patients, may have contributed to the variability in the reported findings.

Duration of DM at the time of renal biopsy was reported to be shorter among patients with NDRD as compared to that in patients with DN superimposed on NDRD, which was broadly accepted as one of the hallmarks of NDRD in a previous literature review. Our results are in agreement with those of previous studies [6–8]. 40.3 % of the entire study population received insulin treatment.

In this study, the NDRD patients had a higher rate of baseline proteinuria (>90 %) and a lower rate of NS (42.3 %); however, the incidence rates in NDRD group did not differ from other groups, which is contrary to a previous study [7], where the degree of proteinuria in DN patients was higher compared to that in patients with NDRD. Likewise, the lack of between-group differences with respect to hematuria in our study (about 70 %) is not consistent with earlier studies which found degree of hematuria helped to distinguish NDRD from DN [6, 27, 30]. However, on subgroup analysis by degree of hematuria, more NDRD patients had urine RBC ˃ 20/HP although the difference was not statistically significant. Further, in a newly published report, detection of dysmorphic erythrocytes was a stronger correlation of NDRD in T2D as compared to hematuria [31]. It may be better to replace hematuria to distinguish NDRD from DN in the future. In this study, NDRD group had a higher baseline level of eGFR than DN ± NDRD group, which is consistent with the lower rate of renal failure in NDRD group reported earlier [30].

No significant differences were observed with respect to dyslipidemia between DN, NDRD, and DN + NDRD groups; the average total cholesterol levels in patients with DN did not differ from that in other groups, which is in agreement with previous studies [13, 21, 32, 33]. Another finding in the present study was the lack of difference in BMI between the 3 groups, which is in accordance with the results of several studies [26, 29], while two conflicting results have been reported in this respect in the Chinese context [32, 34]. Large prospective studies may help to understand the BMI change in Chinese T2D patients with NDRD ± DN in the future.

The frequency of hypertension in the NDRD group (45 cases, 25.7 %) was significantly lower as compared to that in the DN ± NDRD group, which is consistent with earlier reports [8, 13, 21, 29, 34]. This suggests that absence of hypertension in DM is one of the diagnostic features of NDRD, which may be linked to high dietary intake of salt northeastern China [35, 36]. 41.4 % patients were receiving RAAS blockade therapy at the time of renal biopsy; the proportion of patients receiving RAAS blockade is lower than that reported from previous two studies [26, 37]. This suggests that blood pressure control may be preventing progression of renal disease.

The retrospective study design and small sample size are foremost limitations of our study. Of note, selection bias is inevitable in any biopsy-based study. In general, our results can be applicable to T2D patients who were willing to undergo renal biopsy, the likelihood of which is relatively low in the Jilin Province (<10 %) due to the invasive nature of the investigation and, to some extent, reluctance on the part of nephrologists to perform renal biopsy owing to the associated risk of complications. The present study involved T2D patients only in one medical center of Jilin province, which could result in sampling bias. The pathological diagnoses in the patients included in this study were made by the same pathologist, which could have introduced an element of subjectivity. DR has been classically regarded as the indicator of DN, but the reported DR rate was only 15.4 %, which is another shortcoming that limits the generalizability of our findings. Previous studies have shown an association between low serum complement levels (C3/C4) and NDRD [7]; however, due to lack of data we did not assess this association. Such limitations indicate the need for prospective studies to understand the natural history of NDRD superimposed on DN in China.

In this study, 11.0 % of northeastern Chinese T2D patients had NDRD superimposed on DN. The most common pathological diagnosis was hypertensive renal arteriolar sclerosis. Patients with isolated NDRD tended to have a shorter history of DM and lack of DR. Larger, multicenter randomized prospective studies are therefore needed to confirm preliminary changes in T2D patients in order to distinguish NDRD from NDRD superimposed on DN at an early stage, which will help in initiating specific treatment, and thereby, improving kidney survival and reducing the incidence of chronic kidney disease (CKD).
